# Demographic and clinical profile of idiopathic pulmonary fibrosis patients in Spain: the SEPAR National Registry

**DOI:** 10.1186/s12931-019-1084-0

**Published:** 2019-06-17

**Authors:** Estrella Fernández-Fabrellas, María Molina-Molina, Joan B. Soriano, José Antonio Rodríguez Portal, Julio Ancochea, Claudia Valenzuela, Antoni Xaubet, on behalf of the SEPAR-IPF National Registry, M. Aburto Barrenetxea, I. Alfageme Michavila, E. Bollo de Miguel, E. Cano, A. Casanova Espinosa, D. Castillo Villegas, J. A. Figuerola Mendal, R. García Sevila, J. I. Gaudó Navarro, L. Gómez Carrera, J. M. González, S. Herrera Lara, R. Laporta Hernández, M. Marín González, M. A. Nieto Barbero, K. Portillo, A. D. Romero, R. Sánchez Simón-Talero, J. N. Sancho Chust, J. Sellarés Torres, M. J. Soler Sempere, J. Sauleda Roig, L. Tomás López, M. Villanueva Montes

**Affiliations:** 1 Pulmonology Department, Consorcio Hospital General Universitario, Valencia, Spain; 2 Interstitial Diseases Unit, Respiratory Department, Hospital Universitario de Bellvitge, Carrer de la Feixa Llarga, S/N, 08907 – L’Hospitalet de Llobregat, Barcelona, Spain; 3 Pulmonology Department, Hospital Universitario de La Princesa, Madrid, Spain; 4 Pulmonology Department, Hospital Universitario Virgen del Rocio, Sevilla, Spain; 5 Pulmonology Department, Hospital Clinic, Barcelona, Spain

**Keywords:** Anti-fibrotic treatment, Idiopathic pulmonary fibrosis, National registry, Spain, SEPAR

## Abstract

**Background:**

Little is known on the characteristics of patients diagnosed with idiopathic pulmonary fibrosis (IPF) in Spain. We aimed to characterize the demographic and clinical profile of IPF patients included in the IPF National Registry of the Spanish Respiratory Society (SEPAR).

**Methods:**

This is a prospective, observational, multicentre and nationwide study that involved 608 IPF patients included in the SEPAR IPF Registry up to June 27th, 2017, and who received any treatment for their disease. IPF patients were predominantly males, ex-smokers, and aged in their 70s, similar to other registries.

**Results:**

Upon inclusion, mean ± SD predicted forced vital capacity was 77.6% ± 19.4, diffusing capacity for carbon monoxide was 48.5% ± 17.7, and the 6-min walk distance was 423.5 m ± 110.4. The diagnosis was mainly established on results from the high-resolution computed tomography in the proper clinical context (55.0% of patients), while 21.2% of patients required invasive procedures (surgical lung biopsy) for definitive diagnosis. Anti-fibrotic treatment was prescribed in 69.4% of cases, 51.5% pirfenidone and 17.9% nintedanib, overall with a good safety profile.

**Conclusions:**

The SEPAR IPF Registry should help to further characterize current characteristics and future trends of IPF patients in Spain and compare/pool them with other registries and cohorts.

## Background

Idiopathic pulmonary fibrosis (IPF) is a fatal, chronic fibrosing interstitial pneumonia, of unknown aetiology, which affects primarily adults older than 50 [1–3]. Although there is a great variability in the occurrence of IPF, possibly due to geographic and demographic differences, the most reliable data estimate a prevalence ranging approximately 13–20 per 100,000 inhabitants in women and men, respectively [4]. The IPF mean survival ranges between 2 and 4 years from diagnosis for patients not receiving anti-fibrotic treatment [5]. Some factors have been identified to be associated with poorer prognosis and shorter survival time, such as older age, smoking status (smokers and ex-smokers), lower body mass index, more impaired pulmonary function (mainly on forced vital capacity, FVC, total lung capacity, TLC, and diffusing capacity for carbon monoxide, D_LCO_), radiological findings (usual interstitial pneumonia, UIP), a pattern or greater extent of fibrosis, and the development of acute exacerbations or comorbidities, especially pulmonary hypertension and emphysema [6–10]. The diagnosis of IPF requires the collaboration of a multidisciplinary team of specialists to integrate and interpret complex clinical information [11, 12]. Anti-fibrotic treatments for IPF aim to slow down the disease progression and increase the survival time [13, 14]. To date, there are two effective disease-modifying therapies, pirfenidone and nintedanib [2]. Besides the performance of clinical trials for investigating the efficacy and safety of novel drugs, observational studies from routine clinical practice are also required for understanding the natural course of the disease, and identifying differential patterns of diagnosis and treatments [15–17]. Several national IPF registries have been created worldwide; however published results are still scarce [18–24]. The Spanish Society of Pneumology and Thoracic Surgery (‘*Sociedad Española de Neumología y Cirugía Torácica*’, SEPAR) started in 2012 a National IPF Registry aimed to know the clinical characteristics of IPF patients, procedures for diagnosis, and the evolution of patients in Spain. The primary objective of the present study was to characterize the demographic and clinical profile of IPF patients included in the SEPAR IPF National Registry, regardless of any received treatment.

## Methods

### Study design

This prospective, observational, multicentre and nationwide study involved patients with IPF who were included in the SEPAR IPF National Registry and received any treatment for their disease. A total of 28 public hospitals, widely distributed through Spain, participated in the study by including patients in the Registry. Patients were eligible if confirmed diagnosis of IPF. The diagnosis of IPF was based on criteria from international clinical guidelines [10]. Those cases receiving pirfenidone for at least 12 months were analysed to evaluate treatment effects in the real-world clinical practice. Procedures were in accordance with guidelines established in the Declaration of Helsinki, and with the principles of Good Clinical Practices. We have followed and endorsed the Strengthening the Reporting of Observational studies in Epidemiology (STROBE) guidance for reporting observational evidence [24]. Each participating hospital obtained the ethic approval from the Human Research Ethics Committee.

### Data collection and statistical analysis

All pulmonologists from SEPAR were invited to participate in this IPF Registry. They collected the information during routine visits, and uploaded data to the SEPAR website, up to June 27th 2017 [25]. The first patient included was in January 10th, 2012. Database lock occurred in October 5th, 2017. Continuous variables are expressed as the mean, standard deviation (SD), whereas categorical variables as absolute and relative frequencies (%). Median survival time was determined including the corresponding 95% confidence interval (95% CI). Significant prognostic factors associated with mortality were identified by using a backward Cox regression analysis (Hazard ratio, HR). Survival was analysed by Kaplan-Meier methodology. Variables included in the analysis were: age, FVC (% of predicted) at diagnosis, D_LCO_ at diagnosis, anti-fibrotic treatment (yes/no), proton-pump inhibitors (yes/no), reported comorbidities such as pulmonary emphysema (yes/no) or pulmonary hypertension (yes/no), and smoking habits. The patient comorbidities were reported by each participant and the Charlson comorbidity index was calculated after including the data in the Registry. All statistical procedures were performed by using SAS 9.4 software.

## Results

**Fig. 1:**
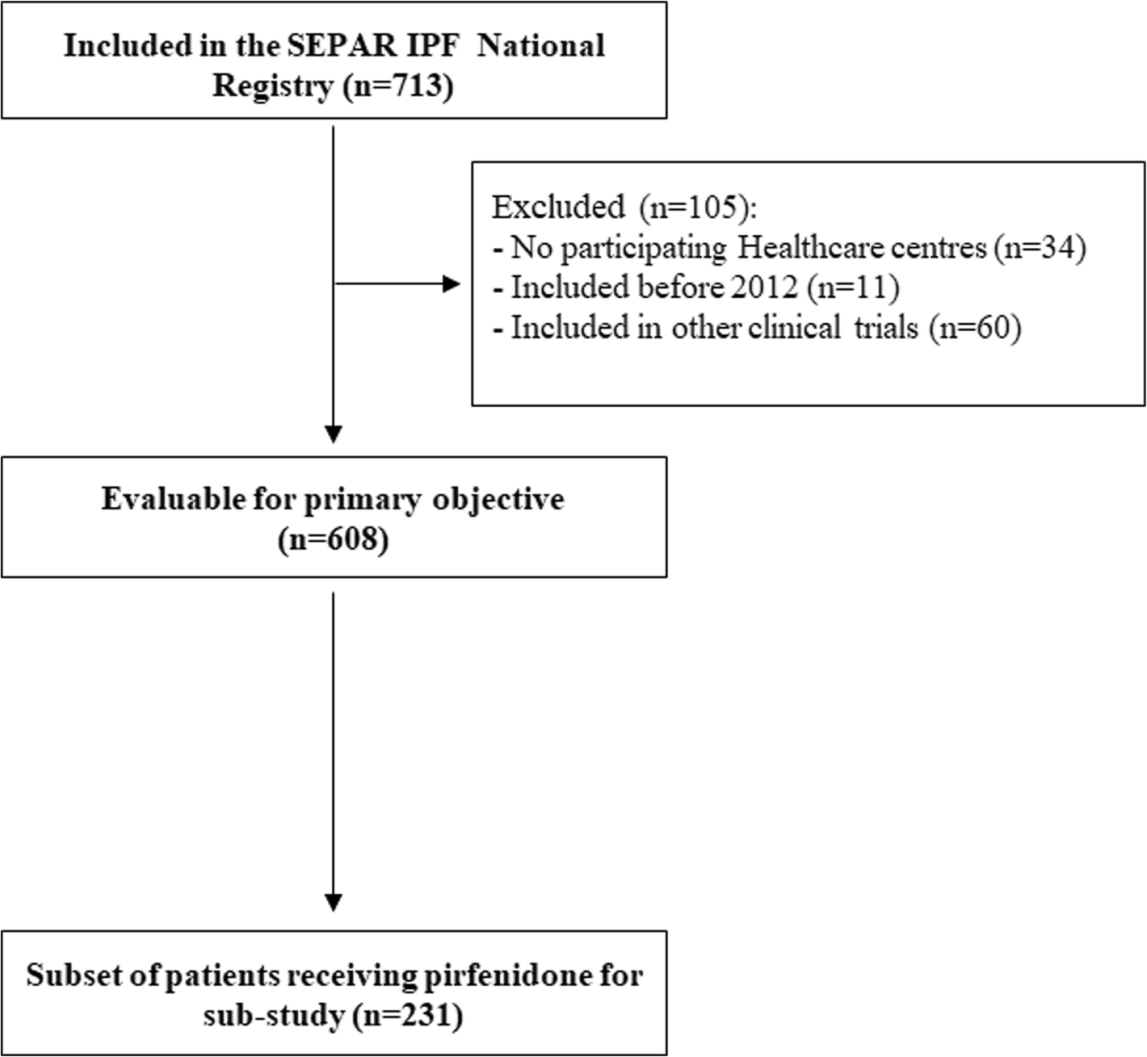
Flowchart of patients. 608 patients were evaluable for the main objective of the study (IPF Spanish patient characterization). 231 patients that received pirfenidone for at least 12 months were analysed to evaluate safety of treatment and clinical features of this subgroup of cases

From 713 patients included in the SEPAR IPF National Registry, 105 were finally excluded (Fig. 1). Therefore, the number of patients evaluable for the primary endpoint was 608. Regarding participating centres, 18 were interstitial lung disease (ILD) academic centres, and 10 non-ILD academic centres [26].

### Registry patients

#### Demographics

Patients were predominantly male (80.8%), with a mean age of 70.2 years (SD 9.2), a mean body mass index of 28.2 kg/m^2^ (SD 4.2), and ex-smokers (63.7%). Demographic and clinical characteristics of patients are shown in Table 1. Time from the onset of symptoms to diagnosis was 20.4 months (SD 21.4).

**Table 1:** Demographic and clinical characteristics of patients with idiopathic pulmonary fibrosis

	N available	Total
Gender (male/female), *n*	608	491 (80.8)/ 117 (19.2)
Age, mean years (SD)	608	70.2 (9.2)
Weight mean kg (SD) ^a^	549	77.0 (14.2)
Height mean cm (SD) ^b^	547	165.0 (8.9)
Body mass index, Kg/m^2^ (SD) ^b^	547	28.2 (4.2)
Smoking habits, *n* (%)	608	
Never-smoker		164 (27.3)
Ex-smoker		382 (63.7)
Smoker		54 (9.0)
Charlson comorbidity index, mean (SD) ^c^	563	3.5 (1.7)
Estimated 10-year survival, mean % (SD) ^d^	607	59.8 (29.6)
Occupational exposure to, *n* (%)	608	
Inorganic particles		131 (23.2)
Organic particles		120 (21.4)
Potentially harmful aerosols		63 (11.3)
Family history, *n* (%)	608	
IPF		57 (9.7)
Other DILD		23 (4.2)
Time from diagnosis to inclusion in the registry, mean years (SD)	608	1.7 (2.2)
Months from the onset of symptoms to diagnosis, mean (SD) ^e^	547	20.4 (21.4)
Main comorbidities, *n* (%)	608	
Diabetes mellitus with no target organ damage		89 (15.8)
Other chronic pulmonary disease		88 (15.6)
Gastroesophageal reflux		74 (12.8)
Pulmonary emphysema		70 (12.1)
Coronary artery disease		50 (8.6)
Myocardial infarction		46 (8.2)
Pulmonary hypertension		36 (6.2)
Malignancies		30 (5.3)
Sleep apnoea-hypopnea syndrome		29 (5.0)
Peripheral vascular disease		24 (4.3)

*SD* standard deviation, *IPF* idiopathic pulmonary fibrosis, *DILD* diffuse interstitial lung disease. Calculated over: ^a^ 549 patients, ^b^ 547 patients, ^c^ 563 patients, ^d^ 607 patients, ^e^ 547 patients

#### Comorbidities

The Charlson comorbidity index was 3.5 (SD 1.7). Diabetes mellitus with no target organ damage (15.8% of patients), chronic respiratory disease (15.6%), arterial hypertension (14%), gastroesophageal reflux (12.8%), pulmonary emphysema (12.1%), and coronary artery disease (8.6%) were the most frequent comorbidities.

#### IPF characteristics

Regarding symptoms or signs indicative of IPF at the time of diagnosis, 89.6% of patients had inspiratory bibasilar crackles, 84.7% dyspnoea (mainly grade 2 or 1), 62.8% non-productive cough, and 29.4% digital clubbing. The mean FVC was 77.6% of predicted (SD 19.4), mean D_LCO_ was 48.5% of predicted (SD 17.7), mean TLC was 72.5% of predicted (SD 16.5), and the 6-min walk distance (6MWD) was 423.5 m (SD 110.4; Table 2).

**Table 2:** Clinical and laboratory findings of patients with idiopathic pulmonary fibrosis

	n available	Total
Pulmonary function tests, mean (SD)		
FVC, L	580	2.6 (0.8)
FVC, % of predicted	584	77.6 (19.4)
FEV_1_, L	574	2.1 (0.6)
FEV_1_, % of predicted	578	81.8 (19.6)
FEV_1_/FVC, % of predicted	569	82.0 (9.5)
Total lung capacity, L	416	4.4 (1.2)
Total lung capacity, % of predicted	451	72.5 (16.5)
D_LCO_ adjusted for haemoglobin	285	8.8 (14.2)
D_LCO_, % of predicted	523	48.5 (17.7)
kCO, % of predicted	458	74.9 (23.1)
PaO_2_, mmHg	215	68.0 (15.8)
PaCO_2_, mmHg	213	38.6 (6.7)
DA-aO_2_, mmHg	213	77.2 (15.7)
Oxygen saturation, %	416	94.9 (2.4)
6-min walking test, mean (SD)		
Distance, m	419	423.5 (110.4)
Autoantibodies in serum, *n* (%)	**500**	**28 (5,6)**
Anti-dsDNA	482	27 (5.6)
Rheumatoid factor	500	18 (3.6)
ANCAs	500	10 (2.0)
Anti-Sm	437	7 (1.6)
Anti-SCL-70	429	3 (0.7)

*SD* standard deviation, *FVC* forced vital capacity, *FEV*_*1*_ forced expiratory volume in 1 s, *D*_*LCO*_ diffusing capacity for carbon monoxide, *kCO* carbon monoxide transfer coefficient, *PaO*_*2*_ partial pressure of arterial oxygen, *PaCO*_*2*_ partial pressure of arterial carbon dioxide, *DA-aO*_*2*_ difference in the alveolar-to-arterial O_2_ gradient, *anti-dsDNA* anti-double-stranded DNA, *ANCAs* anti-neutrophil cytoplasmic antibodies

#### Diagnostic procedures

The high-resolution computed tomography (HRCT) was the most frequent procedure performed at diagnosis (99.2% of patients), followed by pulmonary function tests (98.3%), autoimmune serology (91.8%), chest radiography (81.7%), and the 6-min walking test (71.2%). The confident radiological UIP pattern was found in 65.4% of cases (Table 3). The definitive diagnosis of IPF was established in the 55% of cases by the results of the HRCT in the proper clinical context (after evaluation by ILD clinical and radiological experts). A multidisciplinary discussion with the whole ILD committee was required for definitive IPF diagnosis in 45% of cases; 21.2% of patients that underwent surgical lung biopsy (surgical or transbronchial cryobiopsy), and 23.8% without biopsy.

**Table 3:** Results of different diagnostic procedures regarding usual interstitial pneumonia (UIP) pattern

	Confident UIP pattern	Probable UIP pattern	Indeterminate UIP pattern	Alternative or no suggestive
HRCT	65.4 (391/598)	25.3 (151/598)	7.7 (46/598)	1.6 (10/598)
		Possible UIP	Probably UIP	
Surgical lung biopsy	85.5 (135/158)	8.2 (13/158)	2.5 (4/158)	0.6 (1/158)
Lung cryobiopsy	51.2 (21/41)	14.6 (6/41)	9.8 (4/41)	12.2 (5/41)

*HRCT* high-resolution computed tomography, *UIP* usual interstitial pneumonia

Data are presented as percentage of patients (n/ N available)

#### Treatment approach

Patients were receiving pirfenidone (51.5%) or nintedanib (17.9%) as disease-modifying therapies for IPF at inclusion in the Registry. Main concurrent treatments were: proton-pump inhibitors (68.9%), oxygen therapy (21.5%), or oral corticosteroids (17.8%). From 30.6% of cases not anti-fibrotic treatment: 8.5% were > 85 years, 43.5% presented an FVC > 80%. Of those treated patients, 24.7% experienced at least one adverse event, such as gastrointestinal discomfort (14.0% of patients), anorexia/weight loss (5.9%), alteration of liver enzymes (3.3%), and photosensitivity (2.6%). The adverse event (AE) was the reason for discontinuing the treatment in 27 patients (4.4% of total): pirfenidone (*n* = 15), nintedanib (*n* = 11), or oral corticosteroids (*n* = 1). Recommended non-pharmacological treatment such as rehabilitation and lung transplant were performed in 10.1 and 3.1%, respectively.

#### Survival

**Fig. 2:**
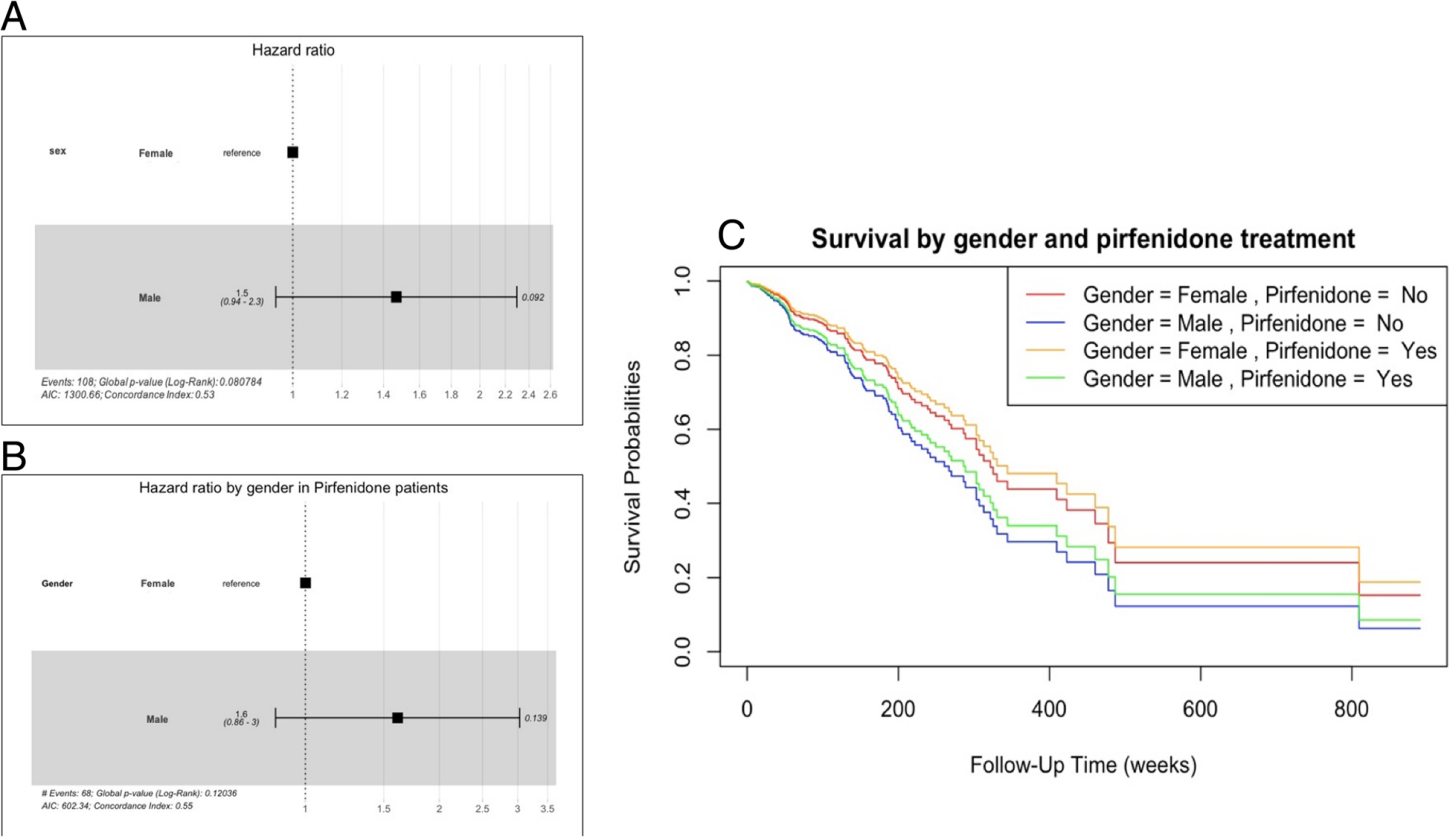
Mortality and survival analyze depending on gender and pirfenidone treatment. **a** No statistically significant higher mortality was observed in males (HR 1.5; 95% 0.94–2.3, *p* = 0.092). **b** There was no statistically significant difference in mortality among pirfenidone treated patients depending on gender (HR 1.6; 95% 0.86–3, *p* = 0.139. **c** Survival time (weeks) analyzed by gender and pirfenidone treatment was not statistically different

A total of 108 patients (17.8%) died during the follow-up, 88 male (81.48%) and 20 females (18.51%) (HR 1.5; 95% 0.94–2.3, *p* = 0.092) (Fig. 2a). The causes of death were: disease progression (45.4%), disease exacerbation (15.7%), lung cancer (5.6%), post-lung transplantation (3.7%), and others /unknown (29.6%). The median survival time was 5.8 years (95 CI 4.8–6.6) since diagnosis. The D_LCO_ at diagnosis was the only prognostic factor associated with mortality (HR 0.609; 95%CI 0.525–0.706). A patient had 39.1% lower risk of death per 10 units of D_LCO_ (%) increased.

#### Patients receiving pirfenidone

A total of 231 patients received pirfenidone for at least 12 months. Patients were predominantly male (79.7%), with a mean age of 68.2 years (SD 9.2), and ex-smokers (68.4%; Table 4). In these patients, the definitive diagnosis was established by results of the HRCT (52.2%), after undergoing the surgical lung biopsy (27.6%), or by multidisciplinary discussion (20.2%).

**Table 4:** Demographic and clinical characteristics of patients with idiopathic pulmonary fibrosis receiving pirfenidone

	N available	Total
Gender (male/female), *n* (%)	231	184 (79.7)/ 47 (20.3)
Age mean years (SD)	231	68.9 (8.4)
Smoking habits, *n* (%)	231	
Never-smoker		59 (25.9)
Ex-smoker		156 (68.4)
Smoker		13 (5.7)
Time from diagnosis to inclusion in the registry, mean years (SD)	231	1.7 (2.1)
Disease progression since onset of symptoms, mean months (SD)	211	22.7 (23.7)
Time of treatment with pirfenidone, mean years (SD)	231	1.5 (1.1)
FVC, mean % of predicted (SD)		
At baseline	226	74.1 (15.5)
After 12 months of treatment	181	71.5 (16.7)
D_LCO_, mean % of predicted (SD)		
At baseline	200	47.4 (16.9)
After 12 months of treatment	150	47.2 (17.6)
6-min walk distance mean m (SD)		
At baseline	158	425.5 (114.7)
After 12 months of treatment	55	429.0 (117.4)
Diagnostic procedures, *n* (%)	231	
High-resolution computed tomography		229 (99.6)
Pulmonary function test		228 (98.7)
Autoimmune serology		213 (94.2)
6-min walk test		173 (77.2)
Surgical lung biopsy		78 (34.2)
Lung cryobiopsy		14 (6.4)
Adverse events related to pirfenidone, *n* (%)	231	54 (23.4)
Gastrointestinal discomfort		22 (9.5)
Anorexia/weight loss		18 (7.8)
Photosensitivity		13 (5.6)
Fatigue		10 (4.3)
Dizziness		3 (1.3)
Alteration of liver enzymes		2 (0.9)
Others		9 (3.9)

*SD* standard deviation, *FVC* forced vital capacity, *D*_*LCO*_ diffusing capacity for carbon monoxide

#### Changes in IPF characteristics

Patients receiving pirfenidone showed a stable lung function in FVC (71.5% of predicted, SD 16.7) and D_LCO_ (47.2% of predicted, SD 17.6) after 12 months of treatment (compared with baseline, 74.1% of predicted, SD 15.5 for FVC; and 47.4% of predicted, SD 16.9 for D_LCO_). The mean 6MWT distance was similar after 12 months of treatment (429.9 m, SD 117.4) than at baseline (425.5 m, SD 114.7).

#### Safety profile

Of patients receiving pirfenidone, 23.4% experienced at least one adverse event (Table 4). Of 231 patients receiving pirfenidone, 15.2% had to modify the treatment during the follow-up period. Reasons of treatment modification (dose reduction *n* = 10, discontinuing the treatment *n* = 9) were as follows: clinical worsening of disease (3.5%), AEs (2.2%) and requiring concomitant medications (1.3%).

#### Survival

Eight patients receiving pirfenidone (3.5%) died during the first 12-month period of treatment. A total of 55 patients (23.8%) died during the follow-up. 87.3% were male and 12.7% female (Fig. 2b). The median survival time was 5.8 years (95 CI 4.2–9.2) since diagnosis (Fig. 2c). Causes of death were: disease progression (32 patients, 58.2%), disease exacerbation (8 patients, 14.5%), lung cancer (5 patients, 9.1%), and others /unknown (10 patients, 18.2%).

## Discussion

Limited information is available about the demographic and clinical profile of IPF patients in Spain, the diagnostic decision-making, and treatments for IPF in real-life setting. To our knowledge, data from 7 national IPF registries have published so far [18–24]. While some IPF features are common in all countries such as male predominance, mean age, and smoking history, other demographic and clinical data differ from other registries, especially mean FVC, D_LCO_ and 6MWD at inclusion, or the basis for the final diagnosis (Table 5). Probably, the heterogeneity of data among registries would depend on the different methodology and type of centres. Some authors have thus suggested creating a global IPF registry, or connecting current IPF networks, such as the ARIANE-IPF pan-European IPF registry and biobank [28]. The goal of the present study is to publish for the first time results from the Spanish IPF national registry on the profile of patients with IPF in routine clinical practice.

**Table 5:** Main sociodemographic and clinical characteristics of patients from other National Registries

	SEPAR IPF National Registry	INSIGHTS-IPF [17]	Finnish IPF [18]	Swedish IPF [19]	Indian IPF ^Ω^ [20]	Australian IPF [21]	EMPIRE IPF [22]	EurIPFreg [23]
Country	Spain	Germany	Finland	Sweden	India	Australia	Czech part	Europe ^b^
Number of patients, n	608	502	111	71	148	647	514	525
Males, %	80.8	77.9	60.4	70.4	73.6	67.7	69.8	73.7
Age mean years	70.2	68.7	73.5	70.0	64.7	70.9	67.0	68.1
BMI mean Kg/m^2^	28.2	27.6	28.1	27.0	–	28.7	28.7	27.2
Ex-smoker, %	63.7	60.2	45.9	56.4	–	71.7	–	65.4
6MWD, mean m	423.5	267.6	–	–		420		388
FVC, mean % of predicted	77.6	72.2	80.4	72.3	57.5	81.0	80.0	68.4
D_LCO_, mean % of predicted	48.5	35.5	57.3	52.1	–	48.4	45.6	42.1
Symptoms indicative of IPF at diagnosis, %								
Dyspnoea	84.7	85.9	44.7 ^a^	–	–	–	–	90.1
Inspiratory bibasilar crackles	89.6	79.0	–	–	–	–	–	95.5
Cough	62.8	74.9	46.6 ^a^	–	–	–	–	53.2
Procedures for definitive diagnosis, %								
HRCT	99.2	90.2	–	72	–	–	–	–
Surgical lung biopsy	26.5	34.1	–	14	–	–	–	32 → 8 ^c^
Multidisciplinary discussion	23.8	21.8	–	20	–	–	–	–

^a^ From patients with available data; ^b^ EurIPFreg, the European IPF Registry has collected information of hospitals from Germany, France, United Kingdom, Italy, Spain, Hungary, and Czech Republic; ^c^ 32% in 2009 and 8% in 2016; ^Ω^ IPF patients are part of the interstitial lung disease registry completed in India

*SD* standard deviation, *BMI* body mass index, *6MWD* 6-min walk distance, *FVC* forced vital capacity, *D*_*LCO*_ diffusing capacity of the lung for carbon monoxide, *HRCT* high-resolution computed tomography

In agreement with international consensus, in most cases the diagnosis was based on typical HRCT images in the clinical context [11, 12, 29–32]. 45% of cases required a case-discussion by the whole ILD multidisciplinary committee. Walsh and colleagues showed a good agreement for the IPF diagnosis between pulmonologists, independently of the type of centre (academic or non-academic centres), with higher concordance in those cases with ILD MDT availability (32). The Fleischner Society recently stated that a confident IPF diagnosis can be achieved when HRCT shows a typical or probable UIP pattern [12]. On the other hand, the MDT discussion of each potential IPF case with probable, possible or inconsistent UIP pattern is recommended in the updated IPF guideline (11). Once made the diagnosis of IPF, the treatment with anti-fibrotics should start as soon as possible [33]. In our study 51.5 and 17.9% of the participants were receiving pirfenidone or nintedanib, respectively. Besides this, some pulmonologists seem reluctant to treat patients with “mild” or “stable” disease, and thus they perform a wait and see approach, probably for avoiding potential AEs or due to misunderstanding by pulmonologists [34, 35]. An international survey revealed that only 40% of patients with a confirmed diagnosis will receive anti-fibrotic treatment; and among untreated patients, 45% receive no treatment at all [34]. Another survey has recently shown that pulmonologists who initiated the anti-fibrotic treatment after more than 4 months of patient’s diagnosis (46% of total) saw fewer patients and had less confidence in the treatment than those who initiated it in ≤4 months [36]. On the other hand, patients with IPF have a higher risk of developing comorbidities [37]. In our study, 12.8% of our patients had gastro-oesophageal reflux, 12.1% pulmonary emphysema, 8.6% coronary artery disease, and 6.2% pulmonary hypertension. It is interesting to note the low number of cases of gastro-oesophageal reflux or cardiovascular disease, compared with literature. Previous studies have shown a prevalence of 87 and 66% for gastro-oesophageal reflux and coronary artery disease in IPF, respectively [38, 39]. Some studies have demonstrated an association between decreased disease progression and longer survival time and the treatment of gastro-oesophageal reflux with antacid [40]; whereas other have not so [41]. In our study, the high percentage of patients receiving proton-pump inhibitors (65.3%) does contrast with the low percentage of patients diagnosed with symptomatic gastro-oesophageal reflux. One explanation is that these treatments were prescribed at the time of IPF diagnosis, before the beginning of the “anti-fibrotic” era. Furthermore, in our study, approximately one in ten patients had family history of IPF, and 55.9% of patients experienced occupational exposures (inorganic, organic particles, or potentially harmful aerosols). In this line, diverse studies have reported an increased risk of UIP in workers exposed to fumes, metal or organic dust [42, 43].

We aimed to describe pirfenidone in clinical practice because it was the first anti-fibrotic available in Spain (more than 2 years before nintedanib). In our Registry, up to 313 patients (51.5%) received treatment with pirfenidone. Despite the proven effectiveness of pirfenidone [34], when receiving treatment, there is always a subgroup of patients who experience inadequate response to therapy. Patients who continue treatment with pirfenidone after having disease progression by month 6 of treatment have a lower risk of FVC decline or death during the subsequent 6 months of treatment [44]. For this reason, it seems recommendable to maintain the treatment with pirfenidone for, at least, 12 months. In our study 8 patients (3.5%) experienced clinical worsening during the treatment with pirfenidone, and 9 patients (3.9%) discontinued treatment. This percentage of discontinuation is slightly lower than previous studies, such as CAPACITY (7.5 and 5.8% of patients), or ASCEND (16%) [45, 46]. It is interesting to note that in our study the median survival rate of total patients and those receiving pirfenidone was similar (5.8 years). This result is in disagreement with other studies, such as the European Registry (EurIPFreg) which reported a significant improvement in survival rate in patients receiving anti-fibrotic treatment (mean 123.1 months; 83% of cases with pirfenidone and 17% with nintedanib) after 7 years of follow-up, comparing with patients not receiving it (mean 68.3 months) [24]. Although no definitive explanation can be provided, we suppose it is because pirfenidone has been only available to patients with FVC < 80% for a long time in most of Spanish hospitals. This fact might have limited the rate of survival in patients receiving pirfenidone. Finally, D_LCO_ at diagnosis was the only factor significantly associated with mortality. In our study, a patient had 39.1% lower risk of death per 10 units of D_LCO_ (%) increased. The impact of D_LCO_ on survival has already been described in previous studies [47, 48]. In fact, some indices combine D_LCO_ together with FVC (the Gender Age Physiology score) and with forced expiratory volume in 1 s (the Composite Physiological Index) for predicting mortality [49, 50].

Main limitations of our study were intrinsically related to the retrospective nature of data collection in the first participants in the Registry (those diagnosed before 2011). Presumably, no available data would improve the knowledge in management of IPF in clinical practice. For example, we only collected information of treatments at the time of inclusion. There is thus a lack of information regarding when they actually received the treatment or whether or not the patient received a new treatment during the follow-up. Another limitation derived from the heterogeneity of patients (including mild, moderate and severe disease) and some uncertainty associated with the diagnostic process, i.e. integrating information from different healthcare professionals, such as clinicians, thoracic radiologists, and pathologists; with varying degrees of experience; and different sites (university or non-university facilities, with or without access to multidisciplinary team meetings) [32]. Furthermore, we couldn’t identify those cases diagnosed based on disease behaviour (working diagnosis), which probably could be part of the IPF cases without lung biopsy that required multidisciplinary discussion. In this regard, SEPAR has recently created a registry of Spanish hospitals according to level of ILD expertise [27]. Differences in the access to medications among Spanish regions may also contribute to heterogeneity. Another limitation was that the type of centre of recruitment (ILD specialist or non- ILD specialist academic centres) may have biased the results as ILD specialist centres could preferentially enrol patients in clinical trials (an exclusion factor for the present study) or prescribe antifibrotic medication. Regarding the 5.6% (19 male/9 female) of cases with low titter of non-specific positive auto-antibodies, all of them had been evaluated by an expert rheumatologist, excluding the association with connective tissue diseases. Albeit, only pulmonologists from ILD and non- ILD specialist centres recruited the patients. Furthermore, this database was not established to evaluate the safety profile or effectiveness of pirfenidone, thus conclusions given with reference to this should be made carefully. Although a higher number of centres would strength results and conclusions, our cohort of patients is representative of the whole population of patients with IPF in Spain. This valuable information can be used in subsequent studies to build prediction models for Spanish patients with IPF. Another goal of the study is that all patients derived from public hospitals, having the same (free) access to procedures and medications.

## Conclusions

Demographic characteristics of patients from the SEPAR IPF National Registry are in accordance with other national registries. In agreement with international guidelines, the diagnosis is mainly based on HRCT in the proper clinical context. A low percentage of patients require invasive procedures for the definitive diagnosis. The treatment with pirfenidone is generally safe and well tolerated, and most cases do not present disease progression after 12 months. Additional studies, including more patients and centres to the Registry, are required to corroborate these results. This SEPAR IPF Registry should help to further characterize current characteristics and future trends of IPF patients in Spain, and compare/pool them with other registries and cohorts.

## Data Availability

The datasets used and/or analysed during the current study are available from the corresponding author on reasonable request.

## Abbreviations

95% CI: 95% confidence interval
AE: Adverse Event
DL_CO_: Diffusing Capacity for Carbon Monoxide
FVC: Forced Vital Capacity
HR: Hazard ratio
HRCT: The high-resolution computed tomography
ILD: Interstitial Lung Disease
IPF: Idiopathic pulmonary fibrosis
SD: Standard Deviation
SEPAR: National Registry of the Spanish Respiratory Society
STROBE: The Strengthening the Reporting of Observational studies in Epidemiology
TLC: Total lung capacity
UIP: Usual Interstitial Pneumonia
